# A Focus on Inflammatory and Bacterial Biomarkers in Secondary Peritonitis

**DOI:** 10.3390/cells14211653

**Published:** 2025-10-22

**Authors:** Valentino Bezzerri, Lorenza Putignani, Elisabetta Mantuano, Alessandro Polini, Luca Navarini, Marta Vomero, Erika Corberi, Valentina Miacci, Paula Elena Papuc, Vincenzo Schiavone, Gianluca Costa

**Affiliations:** 1 Department of Life Sciences, Health and Health Professions, Link Campus University, 00165 Rome, Italy; l.putignani@unilink.it (L.P.); e.mantuano@unilink.it (E.M.); 2 Cystic Fibrosis Center, Azienda Ospedaliera Universitaria Integrata, 37126 Verona, Italy; 3 Unit of Microbiomics and Unit of Microbiome, IRCCS Bambino Gesù Children’s Hospital, 00146 Rome, Italy; 4 Institute of Nanotechnology, National Research Council (CNR-NANOTEC), 73100 Lecce, Italy; alessandro.polini@nanotec.cnr.it; 5 Rheumatology and Clinical Immunology Unit, Department of Medicine, University of Rome Campus Bio-Medico, 00128 Rome, Italy; l.navarini@policlinicocampus.it; 6 Clinical and Research Section, Rheumatology and Clinical Immunology Unit, Fondazione Policlinico Campus Bio-Medico, 00128 Rome, Italy; m.vomero@policlinicocampus.it (M.V.); erika.corberi@unicampus.it (E.C.); 7 Clinical and Research Unit of Colorectal Surgery, Fondazione Policlinico Universitario Campus Bio-Medico, 00128 Rome, Italy; valentina.miacci@unicampus.it (V.M.); paulaelena.papuc@unicampus.it (P.E.P.); 8 Research Unit of Surgery, Department of Medicine and Surgery, University of Rome Campus Bio-Medico, 00128 Rome, Italy; 9 Department of Advanced Biomedical Sciences, University of Naples “Federico II”, 80131 Napoli, Italy; vincenzo.schiavone@unina.it

**Keywords:** peritonitis, inflammation, organoids, peritoneum-on-chip

## Abstract

Secondary peritonitis is a life-threatening intra-abdominal condition arising from gastrointestinal perforation, chemical injury, or catheter-related infections, characterized by marked heterogeneity in presentation and progression. Major subtypes include stercoraceous peritonitis with fecal contamination, fibrinous peritonitis triggered by bile or gastric contents, peritoneal dialysis-associated infections, and pancreatitis-associated chemical peritonitis. Regardless of etiology, these conditions share profound local and systemic inflammatory responses, contributing to high morbidity and mortality. Biomarkers such as procalcitonin (PCT), interleukin-6 (IL-6), high mobility group box 1 (HMGB1), C-reactive protein (CRP), lipopolysaccharide (LPS), neutrophil-to-lymphocyte ratio (NLR), and neutrophil gelatinase-associated lipocalin (NGAL) have emerged as tools for early diagnosis, subtype stratification, and monitoring of therapeutic response. Their prognostic value is particularly relevant in peritoneal dialysis and postoperative intensive care. Advances in multi-omics, patient-derived organoids, peritoneum-on-chip models, and microbiota profiling are reshaping understanding of peritoneal pathophysiology, revealing cellular heterogeneity, immune-microenvironment interactions, and mechanisms of fibrotic remodeling. Key translational challenges include assessing whether omics-derived signatures can predict the need for early re-laparotomy or the risk of abdominal compartment syndrome. Integration of high-dimensional biomarker profiling with mechanistic and functional studies promises a new era of precision medicine in secondary peritonitis, enabling risk-adapted interventions, complication prevention, and tailored strategies to improve outcomes.

## 1. Introduction

Secondary peritonitis is traditionally defined as inflammation of the peritoneal cavity secondary to the loss of integrity of a hollow viscus or a chemically irritating leakage of biological fluids onto the peritoneum. Despite standardized protocols for resuscitation, antibiotic therapy and source-control algorithms, mortality rates remain as high as 20–35% [1]. A key factor contributing to this outcome is that the broad designation of secondary peritonitis obscures substantial biological heterogeneity. Secondary peritonitis can be categorized into diffuse and localized types, the latter generally characterized by a low burden of inflammation. Among the diffuse forms, fibrinous, purulent, stercoraceous, catheter-related, and pancreatitis-associated variants differ in microbial load, biochemical milieu and immune-tissue crosstalk, all of which modulate disease kinetics and therapeutic windows.

Since the landmark description of bacterial translocation and endotoxemia in fecal peritonitis, the field has moved towards quantitative, biomarker-guided risk stratification. Systemic indices such as procalcitonin (PCT) and interleukin (IL)-6 correlate with organ failure scores in adults and children [2,3], whereas ascitic neutrophil gelatinase-associated lipocalin (NGAL) identifies spontaneous or secondary infection in cirrhotic patients with superior sensitivity over absolute neutrophil count [4]. Complement components C3 and C4 have also been proposed as adjunctive markers to distinguish secondary peritonitis from cirrhotic ascites [5]. Furthermore, secondary prophylaxis improves survival in cirrhotic patients after spontaneous bacterial peritonitis, underscoring the importance of early identification and management [6]. A recent meta-analysis across 18 intensive care trials confirmed that PCT-guided antibiotic discontinuation in intra-abdominal sepsis reduces total exposure by almost 15%, without raising mortality [7].

Extending earlier reports on this topic [8,9,10,11], this review organizes secondary peritonitis by etiology, linking microbial burden, peritoneal tissue biology, inflammatory biomarkers, and surgical strategies. Multi-omics (mNGS/cfDNA, metatranscriptomics, proteo-metabolomics, single-cell) are discussed as rapid diagnostic pathways integrated with antimicrobial stewardship and source-control decisions. Patient-derived organoids and peritoneum-on-chip are leveraged to interrogate host–pathogen crosstalk and to test possible therapeutic interventions. Eventually, we emphasize biomarker kinetics within variant-specific algorithms.

## 2. Tissue Organization of the Peritoneum

The peritoneum consists of a single layer of mesothelial cells overlying a vascular, lymph-rich sub-mesothelial matrix. Mesothelial cells are front-line sentinels releasing IL-6, IL-8, tumor necrosis factor (TNF)-α, CXCL1 and CCL2 after exposure to microbial products (for example lipopolysaccharide, LPS) or chemical irritants [7]. Resident macrophages, dendritic cells and a recently characterized cholinergic macrophage subset can modulate the transition from acute inflammation to resolution through acetylcholine-dependent pathways [12]. During severe insult, mesothelial-to-mesenchymal transition (MMT) converts epithelial-like mesothelial cells into extracellular matrix (ECM)-secreting fibroblast-like cells, promoting cellular adhesion and potentially postoperative intestine obstruction.

Vascular and lymphatic capillaries beneath the mesothelium provide a conduit for both bacterial dissemination and immune-cell influx. Breakdown of the glycocalyx in systemic sepsis further amplifies capillary leak, edema, and impaired lymphatic function. Serum syndecan-1 kinetics predict outcome in abdominal sepsis, reinforcing its role as a marker of endothelial dysfunction [13]. Fibrin deposition, typical of fibrinous peritonitis, may conversely help contain the localized contamination when bacterial infections are limited.

Peritoneal mesothelial cells form a dynamic monolayer capable of initiating robust responses to peritoneal insults, including mediating the clearance of contaminated peritoneal fluid and promoting the formation of fibrinous adhesions that localize pathogens and debris, thereby playing a central role in host defense mechanisms during peritonitis. These cells orchestrate a complex array of effector mechanisms within the peritoneal cavity, effectively containing infection in the absence of immediate surgical intervention. Moreover, the interplay between mesothelial cell activities and surgical strategies such as drainage, lavage, and laparostomy critically influences clinical outcomes by modulating fluid dynamics and adhesion architecture. Importantly, it was already proposed two decades ago that therapeutic advances in severe peritonitis management depend on molecular approaches aimed at selectively modulating mesothelial cell functions to optimize local inflammation and tissue repair [14].

## 3. The Microbiome Role Within the Peritoneum

The microbiome in the peritoneum, in terms of organization and characteristics, is an emerging issue, as the peritoneum has been considered for a long time a sterile environment under physiological conditions. However, recent studies suggest that small amounts of microbes or microbial fragments may be present, especially in pathological conditions or in the context of inflammatory diseases. In peritonitis, the peritoneal cavity hosts diverse, often polymicrobial communities, with complex interactions between aerobic and anaerobic bacteria, as well as fungi. In intra-abdominal infections, the peritoneal microbiome reflects the contaminating intestinal microbiome, with a predominance of enteric Gram-Negative bacteria (*Escherichia coli*, *Klebsiella* spp., *Enterobacter* spp.), anaerobes (*Bacteroides fragilis*, *Clostridium* spp.), and Gram-Positive bacteria (*Enterococcus* spp., *Streptococcus* spp.). Microbes in the peritoneal environment often form biofilms or microbial aggregates around necrotic tissue, catheters (in peritoneal dialysis), or fluid collections (abscesses), providing spatial organization of the bacterial biofilm matrices, protecting microbes from the immune system and antibiotics, and frequently complicating the infectious burden and its resolution. Because of the indigenous immunological repertoire, composed of macrophages, dendritic cells, and lymphocytes, the microbiome interacts with the local immune response, modulating and tampering with the inflammatory process, leading to its containment or termination. The immune response can be shaped by the specific microbial composition and by the presence of virulent rather than opportunistic pathogens [15,16]. Currently, microbiome profiling techniques for studying the peritoneal microbiome vary and are aimed at deep taxonomic and functional characterization. Among them, 16S rRNA sequencing can be used to identify bacterial species, while metagenomics and meta-transcriptomics may be applied to assess microbial function and activity, also including advanced culturomics, defined as high-throughput cell culture of bacteria combined with microbial diversity and phylogeny assessment [17]. Strength of this new way of thinking in microbiology, based on the microbiomics discipline, is that maps of microbiome are culture-independent, allowing us to establish a lot of information on the almost complete set of anaerobic bacteria, or colonic microbial populations, based on 16S rRNA-, whole genome- or metagenomics-based next generation sequencing (NGS). Nevertheless, clinical metagenomics still suffers from limited process standardization. Additional work is needed to develop further consensus recommendations, beyond the currently available International consensus statement on microbiome testing in clinical practice, as has already occurred in other areas of clinical microbiology [18].

## 4. Clinical Variants of Secondary Peritonitis

Diffuse secondary peritonitis denotes a heterogeneous constellation of related clinical situations mostly caused by breaching of the visceral barrier. This section groups the condition into five archetypal variants, namely fibrinous mainly related to peptic ulcer perforation (PUP), purulent either due to colonic or non-colonic perforation, stercoraceous, pancreatitis-associated peritonitis, and catheter-related peritoneal dialysis, highlighting the interplay of different contaminating sources, immune response, biomarker profile, microbiota profiling, and preferred surgical approaches that characterize each phenotype (Table 1) [19,20,21]. Localized, contained peritonitis are usually referred to intra-abdominal abscess formation and is characterized by a milder systemic inflammatory response and a favorable prognosis. The clinical assessment of a patient with intra-abdominal infection, whether diffuse or localized, should incorporate internationally validated scoring systems, including the Sequential Organ Failure Assessment (SOFA) score [22].

### 4.1. Fibrinous Peritonitis

Perforation of the stomach or duodenum exposes the peritoneum to gastric acid along with acidic chyme and digestive enzymes, leading to an initial chemical injury. Although this form of peritonitis is characterized by lower bacterial burden compared with small bowel and/or colonic perforation, bacterial infections are often observable in this condition, especially in case of delayed presentation (>12 h). Upper gastro-intestinal (GI) perforation presenting as acute peritonitis predominantly arises from peptic ulcer disease but also from trauma (blunt or iatrogenic), foreign-body ingestion, malignancy and, rarely, infectious causes. Mesothelial cells orchestrate a fibrin-dominated response, with fibrinolytic activity in the peritoneal fluid typically peaking within 24 h. Patients usually present with localized tenderness and mild systemic signs.

In a systematic review of 18 studies involving 536 patients, management strategies ranged from conservative measures, including bowel rest, antibiotic therapy, and percutaneous drainage, to definitive surgical repair including primary closure, omental patching or more extensive gastric diversion in complex cases. Morbidity was considerable, with surgical site infection (SSI), suture leaks and multiorgan dysfunction, and mortality correlated strongly with diagnostic and treatment delays, ranging from approximately 2.9% to 10%. Prompt imaging, early surgical consultation and tailored intervention were associated with improved survival, highlighting the need for standardized diagnostic and therapeutic algorithms to reduce variability in practice and optimize patient outcomes [10]. In addition, it has been recently reported that PCT > 2 ng/mL on admission predicts the need for relaparotomy after surgery for perforated peptic ulcer with 82% sensitivity [19]. However, biomarkers have been poorly investigated in fibrinous peritonitis to date, highlighting an area that deserves further study. For fibrinous peritonitis due to peptic ulcer perforation, the Boey score (shock, severe comorbidity, surgical delay) is a canonical, well-established predictor of postoperative mortality [23]. Furthermore, the Peptic Ulcer Perforation (PULP) score often shows superior discrimination versus Boey and other general severity scores [24]. Mannheim Peritonitis Index (MPI) may additionally provide a well-established benchmark in PUP [25,26]. Empirical antibiotic regimens rely on combination therapy based on third-generation cephalosporin (e.g., ceftriaxone, cefotaxime) or fluoroquinolone, plus metronidazole, or on monotherapy options such as piperacillin-tazobactam and carbapenems. In the case of serious resistance issues, combination of aminoglycoside plus metronidazole plus ampicillin is requested. Intravenous administration is initially preferred in severe cases, usually for 7–14 days, based on microbiological and clinical response. Sometimes, surgical repair of perforation and peritoneal lavage are critical.

### 4.2. Purulent Peritonitis

Purulent peritonitis is mainly triggered by bile, small bowel content, urine, or limited fecal contamination in colonic perforation.

As is known, intestinal perforations carry a high risk of sepsis-associated morbidity and multi-organ dysfunction. A bowel tear allows intestinal contents to enter the peritoneal cavity, leading to abdominal infections. Right- and left-sided perforations differ in prognosis, and fecal-induced peritonitis mouse models may help elucidate mechanisms related to cecum- and colon-derived perforations, underscoring the role of gut microbiota topography in gastrointestinal and abdominal pathophysiology. In this model, mouse intestinal contents from the cecum or colon were peritoneally injected, bacterial burden was quantified by quantitative PCR, and microbiota profiling was performed through 16S rRNA-based meta-taxonomy. Post-injection assessments included survival rates, blood biochemical indices, histological alterations, cytokine expression, immunological signaling, and multi-organ damage. Cecum-derived contents induced more severe sepsis than colon-derived contents, resulting in shorter median survival and more extensive multi-organ injury. Mice treated with cecum IC showed elevated markers of tissue damage in the liver, heart, and kidneys, which contributed to aggravated disease, activating cGAS-STING and TBK1-NF-κB signaling, and promoting more severe systemic inflammation compared with colon IC. Microbial communities also differed between cecum and colon contents, with the cecum harboring a higher abundance of potentially pathogenic bacteria. These findings suggest that the anatomical site of intestinal perforation influences sepsis severity, with cecum perforations associated with greater bacterial burden and relatively enriched pathogenic taxa compared to the colon. This study provides novel mechanistic insights into peritonitis and sepsis [17]. In the case of perforation, the anatomical site is of critical importance, and this is why the role of the gut microbiota, with its full variability and pathobiont burden, may be fundamental for understanding acute infections that often fail to respond to treatment [1].

Current management may include laparoscopic repair, limited peritoneal lavage, and short courses of antibiotics, while antifungal prophylaxis remains controversial as well as in peptic ulcer perforation. A recent French retrospective multicenter cohort study evaluated empirical antifungal therapy in intensive care unit (ICU) adult patients following abdominal surgery for intra-abdominal infections. The study suggested that early empirical antifungal administration may improve short-term survival in HC-IAI [27,28,29,30]. Both anaerobes, including *Bacteroides fragilis*, *Clostridium perfringens*, *Clostridium septicum*, *Peptostreptococcus* spp., *Fusobacterium* spp., and aerobes, including *Escherichia coli*, *Klebsiella* spp., *Enterobacter* spp., *Proteus* spp., *Pseudomonas aeruginosa* (especially in nosocomial context), *Enterococcus faecalis*, *Enterobius faecium*, *Streptococcus anginosus* group, and more rarely *Staphylococcus aureus* dominate the microbial community in purulent peritonitis [31]. Based on these microbial pathogen infections, antibiotic regimens can be condensed into monotherapy or combination therapy. In particular, monotherapy can be represented by carbapenems such as imipenem, meropenem, which have broad-spectrum coverage, but that can be adjusted also based on culture- and molecular-based specific assays. Combined therapy, instead, is mainly represented by cephalosporins, such as ceftriaxone, combined to metronidazole, or by piperacillin-tazobactam, usually 7–14 days, depending on clinical response and severity. Several scoring systems predict mortality in secondary peritonitis [11]. The most commonly used prognostic score for the prediction of postoperative outcomes is the MPI which takes into account several factors, including the type of exudate. A cut-off of 25 points indicates poor prognosis [25,32]. The anatomical grading system provided by the American Association for the Surgery of Trauma (AAST) for Emergency General Surgery (EGS) procedures correlates increasing perforation severity with worse outcomes [33]. Serum lactate levels with the Acute Physiology and Chronic Health Evaluation II (APACHE II) score was found to provide a good prognostic accuracy in predicting 30-day postoperative mortality in patients with peritonitis caused by GI perforation [34].

### 4.3. Stercoraceous Peritonitis

This variant involves gross fecal spillage originating from colon or distal ileal perforation, appendiceal rupture or leakage of colonic anastomosis. The peritoneal cavity is then infected by facultative anaerobes, Gram-Negative bacteria including Enterobacteriaceae or strictly anaerobes, such as *Bacteroides fragilis*, *Clostridium* spp. or the causing necrosis *Clostridium perfringens*, *Peptostreptococcus* spp., *Fusobacterium* spp., *Prevotella* spp. [1]. For anaerobic coverage, antimicrobial regimens include third-generation cephalosporin such as ceftriaxone or ceftazidime plus metronidazole, while as monotherapy, piperacillin-tazobactam and carbapenems can be exploited. Less used are Fluoroquinolones combined with metronidazole. Usually, initial intravenous treatment is indicated, with duration of therapy adjusted according to clinical and microbiological response. Broad range bacterial immunogens, mostly LPS, peptidoglycan and bacterial DNA trigger a cytokine storm dominated by IL-6, TNF-α and high mobility group box 1 (HMGB1), explaining the rapid progression to septic shock and multiorgan failure. Surgical management commonly entails segmental resection with proximal diversion plus extensive lavage. Empiric broad-spectrum antibiotics should include extended-spectrum beta-lactamase (ESBL)-positive Enterobacteriaceae and anaerobes [1]. In this context, a study conducted on Wistar rats undergoing antibiotic prophylaxis before laparotomy with peritoneal contamination and infection (PCI) revealed that the combinatorial effect of coamoxiclav and G-CSF improved the survival rate by normalizing antimicrobial PMN migratory activity, superoxide production, and systemic TNF-α levels [35]. According to recent guidelines from the Italian council for the optimization of antimicrobial use, effective management of intra-abdominal infections relies on early diagnosis, prompt source control, optimized empiric and targeted antimicrobial therapy based on pharmacokinetic/pharmacodynamic principles, and physiological stabilization. These guidelines clarified that targeted antibiotic courses after adequate source control and culture-driven adjustments improve outcomes, prevent opportunistic infections such as those sustained by *Clostridium difficile*, and curb multidrug resistance [28]. Dumitrascu and colleagues evaluated the prognostic utility of a panel of serum and peritoneal biomarkers, including presepsin, procalcitonin, monocyte chemoattractant protein-1 (MCP-1), HMGB-1, IL-6, IL-8, and IL-10 in 32 patients undergoing emergency surgery for secondary peritonitis due to intestine perforation, acute intestinal obstruction, anastomotic leakage, and ruptured abscesses. A longitudinal quantification of these markers, starting from intraoperatively and after 24 h and 48 h from surgery procedures, was performed. Results indicated that, although peritoneal IL-6, IL-8, IL-10, HMGB-1, and MCP-1 were markedly elevated before surgical interventions, their levels did not correlate with patient outcomes. In contrast, early serum presepsin exhibited strong predictive value for the development of sepsis and septic multiorgan failure. The authors therefore propose presepsin as a promising early risk-stratification tool in secondary peritonitis, therefore propose presepsin as a promising early risk-stratification tool in secondary peritonitis [36]. Besides MPI and APACHE II, the Peritonitis Index of Altona II (PIA II) was developed to evaluate patients with intra-abdominal infections, weighting variables that separate survivors from non-survivors. As regards such prognostic scores in stercoraceous peritonitis due to colonic perforations, in a series of 26 cases, a comparative analysis was performed, observing that APACHE II score greater than 20 strongly predict mortality, whereas scores lower than 10 predict survival, whereas MPI and PIA II discriminated less well. This suggests that APACHE II is the most reliable predictor in colonic perforation [37]. The SOFA score also remains a benchmark for mortality risk in intra-abdominal sepsis.

### 4.4. Pancreatitis-Associated Chemical Peritonitis

Severe acute pancreatitis (SAP) is frequently complicated by secondary infections that exacerbate patient morbidity and mortality, necessitating characterization of causative pathogens and resistance profiles. At present, most data on inflammatory biomarkers in pancreatitis-associated chemical peritonitis derive from animal models. Souza et al. demonstrated that peritoneal lavage significantly reduced serum and peritoneal TNF-α and IL-6 levels while elevating IL-10 in a sodium taurocholate (NaT)-induced rat model. This intervention also attenuated pancreatic COX-2 and iNOS expression, indicating systemic anti-inflammatory benefits [38]. Hong et al. measured IL-1β, IL-6 and TNF-α in ascitic fluid, serum and ileal tissue of NaT-induced SAP rats, finding peak cytokine levels at 12 h post-induction, concurrent with histological injury and reduced ileal tight-junction proteins ZO-1 and occludin [39]. Moreover, it has been reported that catalpol (50 mg/kg) pretreatment before NaT infusion markedly decreased serum amylase, lipase, MPO activity and levels of IL-1β, IL-6 and TNF-α, and inhibited NF-κB activation both in vivo and in vitro, supporting its potential as an anti-inflammatory therapy in chemical peritonitis [40]. TNF-α, IL-6, procalcitonin and CRP levels in serum and peritoneal fluid have been proposed as markers of disease severity useful to guide preclinical therapeutic strategies [41]. In a Chinese clinical study conducted between 2016 and 2018, fifty-five SAP patients with culture-confirmed infections yielded 181 isolates from peripancreatic collections, bloodstream, respiratory, urinary, and biliary sites. Gram-Negative bacteria predominated (54.1% of isolates), led by *Acinetobacter baumannii*, *Pseudomonas aeruginosa*, *Klebsiella pneumoniae*, *Escherichia coli*, and *Stenotrophomonas maltophilia*. Gram-Positive organisms accounted for 32% of isolates (principally *Enterococcus faecium* and *Staphylococcus* spp.). Fungi, including *Candida* spp. comprised 13.9%. Bloodstream infections were most prevalent (36.5%), followed by peripancreatic infections (32%). Antibiograms revealed elevated resistance rates among Gram-Negative pathogens relative to national benchmarks, with multidrug resistance especially evident in *Acinetobacter baumannii*. Mortality analysis identified significant associations with older age, prolonged ICU hospitalization, *Acinetobacter baumannii* infection, and urinary involvement. Candidemia also contributed to adverse clinical outcomes, underscoring the need for integrated antifungal stewardship alongside antibacterial therapy. Secondary peritonitis in SAP demands rapid source control and early, pathogen-directed antimicrobial therapy, particularly against multidrug-resistant Gram-Negative organisms, in order to reduce morbidity and mortality [42]. Empirical antibiotic therapy includes Carbapenems, piperacillin-tazobactam or third-generation cephalosporins such as ceftriaxone plus metronidazole. In severe or hospital-acquired infections, coverage for MRSA or fungal infections is indicated and intravenous administration is preferred through a period of 10–14 days, varying depending on clinical progress and surgical findings. However, in some cases, surgical intervention to repair perforation and draining is required. Risk assessment should use pancreatitis-specific tools. The bedside index of severity in acute pancreatitis (BISAP) and its modified version (mBISAP) accurately predict early mortality and organ failure, with performance comparable to APACHE II yet easier bedside use [43,44]. While Ranson’s criteria and APACHE II remain useful [45], BISAP and SOFA are increasingly favored as a practical early-risk tool.

### 4.5. Catheter-Related Peritonitis

Peritoneal dialysis (PD) predisposes to peritonitis via touch contamination, exit-site infection or transmural migration. Gram-Negative bacteria are minimally associated with this secondary peritonitis, despite the infection is triggered by enteric contamination or unappropriated hygiene, such as catheter-related contaminants (i.e., *Pseudomonas aeruginosa*) or nosocomial opportunistic pathogens (i.e., *Enterobacter* spp., *Proteus* spp., *Citrobacter* spp.). Gram-Positive bacteria are the most frequent bacteria, often associated to skin microbiota, through catheter contamination. Main implied bacteria are: the coagulase-negative *Staphylococcus epidermidis*, *Staphylococcus aureus* with serious outcomes, *Streptococcus viridans*, *Streptococcus mitis*, rarely *Enterococcus* spp. [46]. Amongst the others, *Pseudomonas aeruginosa* and the coagulase-negative Staphylococci and are the most representative bacteria for these infections [47]. Indeed, biofilm formation on the catheter internal surface, abundant extracellular DNA and host fibrin can shield microbes from antibiotics, leading to antibiotic resistance and treatment failure [48]. Comparative genomics of *Pseudomonas aeruginosa* isolates from catheter-related peritonitis identified virulence determinants associated with treatment failure [49]. Empirical antibiotic therapy may imply vancomycin or first-generation cephalosporin such as cefazolin, supplemented with aminoglycoside or third-generation cephalosporin, such as ceftazidime. Alternatively, vancomycin combined with ceftazidime can be used as first-line treatments. In case of fungal infections, early catheter removal is strongly recommended. Systemic antifungal therapy should be initiated and continued for approximately 2–3 weeks, tailored to microbiological and clinical response. Biomarkers such as peritoneal NGAL and effluent lactic acid now inform the decision to remove rather than maintain the catheter. NLR, together with platelet-to-lymphocyte ratio (PLR), and systemic immune-inflammation index (SIRI) have been described as independent risk factors for the occurrence of peritoneal dialysis-associated peritonitis (PDAP) [20]. NLR, PLR, and SIRI have been therefore reported as useful diagnostic markers for PDAP since their combined utilization can enhance diagnostic accuracy. Beyond acute management, recurrent PDAP predisposes to long-term sequelae such as encapsulating peritoneal sclerosis (EPS), a severe, late complication marked by fibro-adhesive encapsulation and bowel obstruction. Multi-omics profiling is refining pathogenesis and risk stratification in this case (see also Section 8). Single-cell and bulk transcriptomics can reveal mesothelial-to-mesenchymal transition programs and profibrotic signaling (TGF-β/SMAD, Wnt/β-catenin), alongside expansion of inflammatory macrophage and activated fibroblast subsets expressing elevated levels of type I collagen (*COL1A1*). Proteomic and metaproteomic analyses of peritoneal fluids show ECM remodeling and angiogenic signatures that indicate peritoneal membrane failure. Metabolomics and lipidomics identify oxidative stress fingerprints associated with microvascular damage and progressive ultrafiltration loss. Small-RNA and exosome cargo (e.g., dysregulated miR-21 and miR-29) correlate with fibrosis burden and may serve as minimally invasive surveillance tools. In PDAP, prognosis is better estimated with dialysis-specific nomograms and multivariable risk scores predicting treatment failure, refractory infection, or catheter loss. These models showed reliable calibration and discrimination parameters in multicenter cohorts [50]. The NLR is generally independently associated with poor outcomes and is incorporated into some PDAP scoring models [51].

### 4.6. Localized Peritonitis: Covered Bowel Perforation and Appendicitis

Covered (or contained) perforation refers to a minimal bowel tear that is rapidly sealed by the omentum or adjacent viscera, producing localized peritoneal contamination rather than free fecal spillage. Covered perforation peritonitis represents a localized inflammatory state in which timely clinical examination and risk stratification, along with CT, play a pivotal role, allowing conservative or minimally invasive source control procedures [52]. Canonical examples include abscess formation in low-grade diverticulitis, such as Hinchey-Wasvary Ia-Ib, and appendicitis. In these instances, non-surgical management is often feasible by employing nasogastric decompression, treatment with proton-pump inhibitors, broad-spectrum antibiotics, and close monitoring. By contrast, failure to improve within the first 12–24 h, onset of sepsis, or radiologic signs of ongoing contamination warrant surgical control. For colonic contained perforation, international guidelines recommend antibiotics with or without percutaneous drainage for diverticulitis. In the case of appendicitis with abscess, initial non-operative management is reasonable, while the need and timing of interval appendectomy remain debated [53]. Elevated CRP levels (around or above 150 mg/L) are associated with complicated presentations, increasing the probability of abscess and perforation, therefore supporting invasive management [54]. Higher NLR levels are associated with complication risk in acute diverticulitis and can complement CRP when deciding between conservative versus surgical or percutaneous procedures in contained perforations [55]. The MPI assigns substantially lower weights to localized contamination and non-stercoraceous exudate [26,56]. Integrating CRP (with attention to high-risk thresholds), NLR, and a validated prognostic system such as the MPI provides a pragmatic framework to identify the subset of covered perforations that warrant early intervention despite initially muted clinical signs. Piperacillin-tazobactam or third-generation cephalosporins plus metronidazole or Carbapenems can be requested for severe or high-risk cases. In less severe cases, even when initial intravenous therapy is required, step-down to oral antibiotics may be considered once the patient stabilizes. Nevertheless, drainage of any localized abscess (percutaneous or surgical) may be necessary, with ongoing clinical and laboratory monitoring to guide therapy adjustments.

## 5. Pathophysiology Across Variants

**Figure 1:**
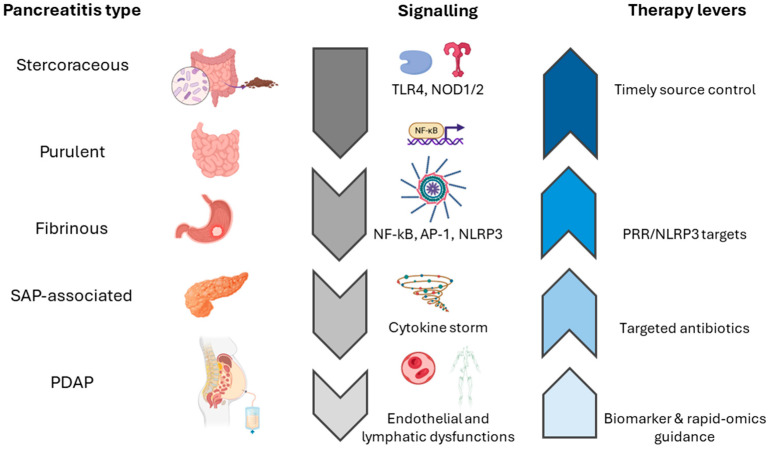
Comparative mechanisms and therapy levers in secondary peritonitis. Stercoraceous peritonitis is driven by massive microbial load, leading to TLR4 and NOD1/2 stimulation; cytosolic LPS activates NF-κB, caspase-11, and NLRP3, inducing IL-1β/IL-18 release, pyroptosis, vascular leak, finally unleashing cytokine storm; neutrophils are recruited through IL-8 signaling, degranulate uncontrollably, and systemic injury follows. Purulent peritonitis shows high peritoneal TNF-α, IL-8, and neutrophil elastase that fall after source control; serum cytokines remain lower, reflecting compartmentalized inflammation, while antibiotics and macrolide immunomodulation can reduce chemotaxis and ROS. Fibrinous peritonitis features NF-κB and AP-1 co-activation in mesothelium, promoting IL-8, MMP-9, mesothelial-to-mesenchymal transition, and fibrin deposition that organizes abscesses. Pancreatitis-derived peritonitis is dominated by inflammasome activation through mitochondrial damage, amplifying IL-1β signaling. In catheter-related peritonitis, biofilm-driven PRR activation is combined with endothelial and lymphatic dysfunction; syndecan-1 indicates glycocalyx injury and predicts outcomes, while nitric oxide-dependent lymphatic dysmotility impairs clearance and sustains chronic inflammation. SAP, severe acute pancreatitis; PDAP, peritoneal dialysis-associated peritonitis; PRR, pattern recognition receptor.

A coherent understanding of how each etiological variant perturbs peritoneal homeostasis, from host–pathogen recognition, through cytokine storm, to vascular, lymphatic and transcription factor signaling, is critical for moving towards personalized medicine approaches and antimicrobial regimens. The following subsections shed light on the convergent mechanisms that unify stercoraceous, purulent, fibrinous, catheter-related, and pancreatitis-associated peritonitis, while pointing out the divergence aspects that may justify variant-specific therapies (Figure 1).

### 5.1. Microbial Load and Pattern Recognition Receptors

The transition from simple peritonitis to severe sepsis is mediated by overwhelming engagement of pattern recognition receptors (PRRs). In a murine cecal ligation and puncture (CLP) model, Zhou et al. demonstrated that simultaneous stimulation of TLR4 with LPS and NOD1/2 with Tri-DAP and MDP produces a massive NF-κB activation, markedly elevating TNF-α and IL6 mRNA transcription and potentiating NLRP3 assembly, amplifying the innate response to polymicrobial sepsis [57]. Hagar et al. showed that high peritoneal loads of Gram-Negative bacteria allow cytosolic entry of LPS into macrophages, directly activating caspase-11. This non-canonical pathway complements TLR4-MyD88/TRIF-mediated priming by triggering gasdermin D cleavage, pyroptosis, and robust IL-1β and IL-18 release [58]. Oh et al. further demonstrated that NOD2 recognition of peptidoglycan drives NF-κB-dependent induction of pro-IL-1β and establishes an IL-1β/IL-10 feed-forward loop in peritoneal neutrophils. IL-10-mediated upregulation of complement C5a generation then amplifies neutrophil activation and cytokine secretion in a self-sustaining circuit [59]. Srdić et al. synthesized these findings, showing that TLR4 and NOD1/2 saturation in fecal peritonitis leads to maximal NF-κB nuclear translocation, inflammasome assembly, and IL-1β/IL-18 release, driving pyroptotic cell death, vascular leakage, hypotension, and multi-organ dysfunction [60]. Kelley et al. articulated the canonical two-signal model of NLRP3 activation. NF-κB priming downstream of TLR4/NOD1/2 induces NLRP3 and pro-IL-1β, whereas ATP, ion fluxes, or phagocytosed bacterial components trigger inflammasome oligomerization and caspase-1 activation [61]. Mitochondrial Ca^2+^ overload also represents a pivotal activator of inflammasome. In fact, uptake through the mitochondrial calcium uniporter (MCU) is required for NLRP3 assembly and processing of IL-1β and IL-18 into bioactive forms in human bronchial epithelial cells exposed to *Pseudomonas aeruginosa* [62]. Although derived from preclinical studies, these interconnected mechanisms highlight TLR4/NOD signaling, caspase-11, complement C5a, calcium signaling, and NLRP3 as promising targets to interrupt the self-amplifying inflammatory cascade in secondary peritonitis.

### 5.2. Role of Neutrophils

Neutrophils rapidly transmigrate into the peritoneal cavity along an IL-8-mediated chemotactic gradient [63], orchestrated by mesothelial cell-derived chemokines and resident macrophages [64,65]. Once recruited, neutrophils kill bacteria through oxidative bursts and release of antimicrobial peptides such as defensins and cathelicidins, achieving maximal ROS flux in rodent peritonitis models [66]. In localized secondary peritonitis, this focused recruitment promotes organized abscess formation via neutrophil-fibrin interactions, containing infection and preventing systemic spread [67].

In contrast, diffuse fecal peritonitis generates overwhelming bacterial loads that sustain NF-κB activation in peritoneal macrophages and mesothelial cells, resulting in IL-8 hyperproduction. This oversaturates neutrophil effector functions, precipitating uncontrolled degranulation, mesothelial injury, and amplified systemic inflammation [68].

Adjunctive broad-spectrum β-lactams, including ceftriaxone, reduce microbial burden and downregulate peritoneal IL-8 and C5a, decreasing neutrophil chemotaxis by 40–60% in rodent models [68]. Macrolides, such as azithromycin, exert immunomodulatory effects by inhibiting CD11b/CD18 upregulation and ROS release, limiting tissue damage [69]. Early administration of NSAIDs attenuates prostaglandin-mediated vascular permeability and neutrophil extravasation but may impair bacterial clearance under high-inoculum conditions [70]. Glucocorticoids, such as dexamethasone, markedly inhibit NF-κB-dependent chemokine secretion, reducing peritoneal neutrophil counts in rat models, although at the risk of exacerbating sepsis progression [71].

### 5.3. Cytokine and Chemokine Profiles

Comparative analyses of cytokine kinetics in secondary peritonitis and sepsis reveal distinct pro- and anti-inflammatory patterns with prognostic and mechanistic relevance. In a prospective cohort of 92 critically ill abdominal sepsis patients, serum IL-6 peaked sharply within 24 h (mean 217 ± 48 pg/mL) before maximal SOFA scores on day 2 [72]. Similarly, in 57 patients meeting SIRS/sepsis criteria, IL-6 peaked on day 1 (mean 153 ± 33 pg/mL) and correlated with APACHE II scores [73], establishing IL-6 as an early biomarker of organ dysfunction independent of peritoneal insult type.

In patients undergoing planned relaparotomy for severe fecal peritonitis, peritoneal fluid cytokine concentrations exceeded serum levels. IL-6 cleared only from serum, while IL-8 remained elevated in both compartments. TNF-α and IL-1β were low in serum but variable in peritoneal fluid [74], suggesting differential clearance kinetics within the first 48 h. Pre-operative levels of TNF-α, IL-8, and neutrophil elastase (NE) in 21 patients with purulent peritonitis were markedly higher in peritoneal exudate than plasma; standard surgical procedures reduced these intra-abdominal concentrations to approximately 10% of baseline [75]. TNF-α levels correlated with microbial load, whereas IL-8 and NE did not, and neither predicted postoperative mortality. TGF-β in peritoneal fluid correlated with subsequent adhesion formation, serving as a surrogate for mesothelial activation and fibroblast recruitment [40]. Spatial transcriptomics of peritoneal biopsies could identify discrete chemokine niches at the mesothelial-submesothelial interface, implicating CXCL and CCL gradients in leukocyte compartmentalization and adhesion formation.

### 5.4. Transcription Factor Signaling

NF-κB activation is a common feature in secondary peritonitis. PRR engagement, including TLR4 and NOD1/2, converges on the IκB kinase (IKK) complex; IKKβ phosphorylates IκBα, targeting it for degradation and releasing p65/p50 NF-κB dimers to the nucleus, inducing transcription of IL-6, IL-8, TNF-α, and CXCL1 [76]. In a murine CLP model, delayed IV administration of 1 mg/kg IKK16 (IKKβ inhibitor) reduced IκBα phosphorylation by 45% and p65 nuclear translocation by 50%, decreasing TNF-α and IL-6, preserving cardiac function, reducing pulmonary MPO activity, and increasing 72-h survival from 20% to 60% [76].

Activator Protein-1 (AP-1), mainly c-Jun/c-Fos heterodimers, is activated downstream of MAP kinase signaling in mesothelial cells. Under hyperosmotic conditions, TGF-β1 stimulation upregulates matrix metalloproteinase (MMP)-9 up to three-fold, promoting ECM remodeling [77]. AP-1 cooperates with NF-κB on shared promoters, enhancing IL-8 expression up to 2.5-fold in response to inflammatory or hypoxic stimuli [78]. In vitro silencing of c-Jun reduces mesothelial-to-mesenchymal transition markers (e.g., fibronectin) and fibrin deposition by 60% in bile-induced peritonitis models, underscoring the critical role of AP-1 in peritoneal fibrosis [79].

### 5.5. Vascular and Lymphatic Dysfunction

Endothelial glycocalyx degradation, as evidenced by the proteolytic shedding of syndecan-1 into the circulation, serves as a sensitive indicator of barrier disruption and systemic spillover of inflammatory mediators [80]. Elevated plasma syndecan-1 levels correlate with widespread endothelial activation, loss of vascular integrity, and concomitant activation of coagulation and fibrinolysis pathways, reflecting the transition from localized peritoneal inflammation to systemic inflammatory response. Concurrently, chronic peritoneal dialysis-associated peritonitis impairs intrinsic lymphatic contractility through excessive nitric oxide production by inducible nitric oxide synthase, leading to dilatation and reduced frequency of lymphangion contractions. This dysfunction can be noninvasively visualized in vivo using indocyanine-green fluorescence imaging, which reveals delayed clearance and retrograde lymphatic flow within diaphragmatic collecting vessels [81]. Finally, serial measurements of serum syndecan-1 in patients with abdominal sepsis demonstrate that a persistently high or rising syndecan-1 concentration over the first 72 h post-surgical insult predicts worse outcomes, including prolonged organ dysfunction and increased mortality, thereby underscoring its prognostic value as a dynamic biomarker of endothelial injury and capillary leak in the septic milieu [82].

## 6. Inflammatory Biomarkers

Robust biomarkers bridge complex peritoneal biology and real-time decisions. Table 2 summarizes key markers, sources, utilities and recent updates. Moreover, wearable biosensors capable of continuous cytokine monitoring have been piloted, enabling real-time assessment of peritoneal inflammation at the bedside.

### 6.1. Biomarker Kinetics

Biomarker monitoring may outperform static thresholds in guiding postoperative management of secondary peritonitis. Absolute PCT values at admission predict severity in secondary diffuse peritonitis, identifying cut-offs of 15.3 ng/mL for septic shock and 19.6 ng/mL for mortality in these cases [97]. PCT-guided antibiotic discontinuation protocol was implemented in 121 ICU patients after source control. When PCT fell under 30% at 24 h, antibiotics were safely stopped, resulting in a 50% reduction in treatment duration without increases in mortality or septic complications [98]. However, static clinical and laboratory variables alone can poorly predict the need for relaparotomy. In a survey of 21 potential decision factors, only three were independent predictors, including persistent hemodynamic instability, ongoing vasopressor requirement, and radiologic evidence of leak. Even their combination achieved only moderate accuracy, underscoring the need to integrate dynamic biomarkers and imaging into relaparotomy algorithms. Planning of relaparotomy with an on-demand strategy guided by both early lactate clearance (>15% at 6 h) and PCT kinetics (>30% drop at 24 h) was conducted in a randomized clinical trial. The on-demand group experienced a marked reduction in first-negative relaparotomy rates (31% vs. 66%), shorter hospital/ICU stays, and 23% lower direct medical costs, without compromising mortality or major morbidity [99,100].

### 6.2. microRNA Signature

Small non-coding RNAs, particularly microRNAs (miRNAs), have emerged as pivotal upstream regulators of the cytokine storm in sepsis and promising minimally invasive biomarkers. The expression of miR-146a is up-regulated by NF-κB in response to TLR agonists and directly binds the 3′-UTRs of IRAK1 and TRAF6, establishing a negative-feedback loop that attenuates TLR-mediated signaling [101]. Again, miR-155 is robustly induced in LPS-stimulated macrophages, where it promotes TNF-α expression and amplifies the inflammatory response [102]. High-throughput profiling of peritoneal exudate cells in a murine chronic peritonitis model revealed coordinated dysregulation of more than 30 miRNAs, including miR-155, miR-146a and miR-223, across early (4 h), intermediate (24 h) and late (48 h) time points. These miRNAs converge on the NF-κB pathway, modulating cytokine outputs such as TNF-α, IL-1β and HMGB1, and offering a mechanistic basis for endotoxin tolerance in persistent peritoneal infection [95]. Clinically, profiling of effluent miRNAs from patients with peritoneal dialysis-associated peritonitis revealed that miR-223 levels in the dialysate spike on day 1 of infection and correlate with NGAL, predicting catheter loss with an AUC of 0.79 [95]. Additionally, in PD effluent, exosomal miR-432-5p levels correlated with peritoneal solute transport rate (PSTR), ultrafiltration (UF), and dialytic sodium removal (DSR). Levels of miR-432-5p were higher in high-PSTR patients, positively associated with PSTR, but negatively correlated with 4 h UF and DSR. It has been reported that miR-432-5p can reduce the expression of the epithelial sodium channel α-subunit (α-ENaC) in peritoneal mesothelial cells. Thus, miR-432-5p may serve as a biomarker for impaired fluid and sodium removal in peritoneal dialysis [96]. Using a murine model of cecal ligation and puncture (CLP), it has been described that pharmacological inhibition of miR-155 or miR-146a sustained by antagomir can reduce systemic IL-6 levels, ameliorating organ injury and improving the survival rate by approximately 30% compared with controls. Knockdown of miR-146a via antagomir delivery attenuated cardiac dysfunction in septic mice through de-repression of IRAK1 and TRAF6, underscoring the therapeutic potential of miRNA modulation [103].

## 7. Clinical and Surgical Management

The treatment of secondary peritonitis relies on two fundamental principles: rapid surgical source control even with intraperitoneal negative-pressure therapy and effective antimicrobial therapy [104]. Early surgery, whether by laparotomy or laparoscopy, is crucial both for eradicating the septic focus and for obtaining peritoneal fluid, which serves as a valuable substrate for diagnostic and prognostic analyses. While the choice between planned relaparotomy and on-demand re-exploration remains debated, emerging evidence suggests that biomarker kinetics may eventually support more tailored decisions in this context [11,105,106].

In recent years, inflammatory biomarkers have gained prominence in risk assessment and monitoring. PCT has shown superior performance compared with conventional markers such as CRP and leukocyte count, with high or persistent values associated with adverse outcomes [107]. Pediatric studies further indicate a possible prognostic role for CRP, IL-6, N terminal pro-B-type natriuretic peptide, and calprotectin, underscoring the potential of age-specific biomarker panels [108]. PCT is further increasingly employed to optimize antimicrobial strategies. In fact, since randomized trials confirmed that PCT-guided discontinuation safely reduces antibiotic exposure, this approach has been incorporated into international guidelines [109,110,111,112].

Microbiological investigations, antibiotic selection, and pharmacokinetic considerations complement this biomarker-driven approach. Peritoneal fluid cultures remain indispensable for guiding targeted therapy and de-escalation, while broader laboratory analyses, including cellular and biochemical markers, are being explored for their diagnostic and prognostic value [113]. Bloodstream infection parameters also provide critical insights. For instance, a time-to-positivity of ≤12 h in *Klebsiella pneumoniae* bacteremia secondary to intra-abdominal infection has been described as an independent predictor of mortality, highlighting the prognostic potential of culture kinetics [114].

Moreover, studies of antibiotics in peritoneal fluid demonstrated that standard dosing of meropenem (1 g every 8 h) may underexpose the peritoneal compartment, suggesting that prolonged infusion or higher daily doses are required to achieve adequate time above minimum inhibitory concentration (fT > MIC) [115]. Similarly, several guidelines underscore the importance of optimizing dosing strategies and integrating therapeutic drug monitoring where available [105,106,109,110]. These strategies are particularly relevant in the face of rising antimicrobial resistance, where unnecessary treatment carries both individual and community risks [112,113].

Standardizing biomarker-guided antibiotic therapies for each peritonitis type may reduce adverse events and resistance while preserving efficacy. In case of fecal contamination and distal colonic sources, initial broad coverage against enterobacteria (including ESBL risk), anaerobes, and particularly *Enterococcus* spp. is recommended. Combinations of β-lactam/β-lactamase inhibitor or carbapenems may be useful depending on local epidemiology and severity. Conversely, bile or gastroduodenal leaks with low bacterial burden often permit narrower agents and short courses after effective source control. PDAP should follow intraperitoneal, pathogen-directed antibiotic treatments with early consideration of catheter management as recommended by the International Society for Peritoneal Dialysis (ISPD) guidelines [46]. Among the different phenotypes, duration of treatment should be based on source control rather than syndrome labels. Randomized clinical trials support almost 4 days of therapy after adequate control, even in septic or percutaneously drained cases, without worse outcomes [8]. Metagenomic NGS of peritoneal samples might accelerate pathogen identification (particularly for microorganisms that are difficult to culture) and resistance factors, enabling earlier de-escalation and alignment of antibiotic treatment with the molecular microbiology of each strain.

Early omics assays in peritoneal samples (i.e., 16S/qPCR and host–pathogen targeted panels), therefore before first-dose antibiotics and source control, might provide useful directional indications. Integrating these methods in early timepoints may enable earlier de-escalation or targeted escalation, supporting phenotype-specific decisions. Omics-guided antibiotic treatments may abbreviate courses after effective control of gastrointestinal leaks, or reduce carbapenem regimens when ESBL genes are absent. Despite standardized antibiotic protocols, resistome characterization, based on ecological analysis of the gut microbiome, has become central to mapping bacteria-bacteria and bacteria-fungi interkingdom networks and to delineating pathobiont-mediated colonization dynamics and emergent antimicrobial resistance mechanisms.

Adjunctive measures and supportive therapies further contributed to improved outcomes. Point-of-care evaluation of NGAL in peritoneal drainage fluid has been shown to predict early organ dysfunction [116], while serial lactate clearance was incorporated into an enhanced recovery after surgery (ERAS) pathway and was associated with a 15% absolute reduction in 30-day mortality [117]. Furthermore, early initiation of enteral feeding, within the first 24 h after surgery, has been shown to attenuate systemic inflammation in terms of CRP and IL-6 levels and to shorten ICU stay compared with delayed feeding [118].

Overall, the clinical management of secondary peritonitis is evolving toward a precision-based paradigm, where surgical techniques, microbiological diagnostics, inflammatory and bacterial biomarkers, and adjunctive strategies (i.e., nutrition) converge to refine decision-making and improve patient outcomes.

## 8. Advanced Analytical Techniques

Intestinal and peritoneal organoids and, more recently, peritoneum-on-chip devices, can faithfully recreate epithelial, mesenchymal and vascular compartments, enabling high-throughput drug screening, mesothelial regeneration and dissection of cell-microbe and cell–cell interactions in peritoneal injury and fibrosis. Integrated omics approaches, including single-cell transcriptomics of peritoneal exudates, metagenomic sequencing of cell-free DNA in dialysis effluent, agnostic microbiome profiling and host RNA-seq in murine models, may enhance pathogen and pathobiont detection, antimicrobial resistance profiling, and prognostic modeling, paving the way for precision diagnostics and personalized therapeutics in secondary peritonitis.

### 8.1. Organoids and Peritoneum-on-Chip Models

Intestinal and peritoneal organoids have emerged as sophisticated three-dimensional in vitro models derived from single Lgr5^+^ adult stem cells or human pluripotent stem cells that faithfully recapitulate both epithelial and mesenchymal compartments of their tissues of origin. A single Lgr5^+^ intestinal stem cell can self-organize into crypt-villus structures in matrigel without any exogenous mesenchymal niche, generating organoids that exhibit functional Paneth cells, goblet cells, and enterocytes, as well as physiological Wnt-dependent growth dynamics [119]. According to these findings, human pluripotent stem cells were directed through definitive endoderm and midgut patterning to produce complex intestinal organoids containing differentiated enterocytes, goblet cells, Paneth cells, and subjacent mesenchyme, enabling modeling of epithelial–mesenchymal transitions and host-microbial interactions in gut inflammation and fibrosis [120]. Organoid-enabled high-throughput drug screening has further identified tranexamic acid, a lysine analogue that inhibits plasminogen activation, as an effective antifibrinolytic adjunct in bile acid-induced peritoneal injury models. Tranexamic acid treatment reduced fibrin deposition, attenuated inflammatory cytokine release, and improved mesothelial viability, highlighting its promise for personalized therapy in bile leak peritonitis [121].

Organ-on-chip technology has shown remarkable examples on how biomedical research can emulate different organ functions in vitro with a high level of physiological/pathological fidelity [122,123]. In the field of peritoneum-centered disorders, an “omentum-on-a-chip” model comprising a multicellular, vascularized microfluidic tissue platform that integrates human peritoneal mesothelial cells, adipocytes, endothelial networks, and extracellular matrix to recapitulate key features of the omental tumor microenvironment was reported. This platform may provide a robust in vitro system for dissecting cellular crosstalk during intraperitoneal metastasis and for evaluating therapeutic strategies targeting the vascularized peritoneal niche [124]. Other microfluidic platforms recapitulating the human peritoneum are currently under investigation. A reproducible laparoscopic peritoneal wash cytology (PWC)-based method to isolate and culture primary human peritoneal mesothelial cells. Comprehensive characterization through brightfield and immunofluorescence microscopy, flow cytometry, and Raman microspectroscopy revealed cytokeratin-positive mesothelial populations clearly distinguished from fibroblasts, with variable calretinin and consistent WT-1 expression. Raman imaging and multivariate analysis demonstrated comparable biochemical and morphological profiles between cultured cells and cryo-fixed peritoneal tissue, confirming physiological relevance. This platform supports the generation of 2D, 3D, and microfluidic models of the human peritoneum, enabling investigation of peritoneal pathophysiology and therapeutic testing. By providing accessible patient-derived mesothelial cultures, this approach could improve in vitro systems for studying peritoneal diseases such as inflammation, adhesions, endometriosis, and metastasis [125].

### 8.2. Omics Integration

A recent single-cell RNA sequencing analysis of human peritoneal exudates identified six transcriptionally distinct macrophage clusters, including a ChAT-expressing “cholinergic” subset whose abundance peaks around day 7 of symptom onset, corresponding to the resolution phase of inflammation, and, when these cluster abundance signatures were integrated with 15 baseline clinical parameters in a Random Forest model, achieved an AUROC of 0.86 for predicting a ≥2-point escalation in SOFA score within 72 h of hospital admission, markedly outperforming conventional ΔSOFA metrics [12].

Recent integrative omic investigations have substantially advanced our understanding of secondary peritonitis by combining high-throughput pathogen detection with comprehensive host-response profiling. Unbiased metagenomic sequencing of cell-free DNA extracted from peritoneal dialysis effluent of 50 patients with clinically confirmed peritonitis versus 30 uninfected controls was performed, demonstrating that cfDNA sequencing identified a median of 3.5 pathogens per sample and detected viral and fungal organisms undetected by standard culture. Notably, 25% of culture-negative episodes yielded identifiable pathogens via cfDNA, resulting in a 40% increase in diagnostic sensitivity over culture alone [126]. Importantly, cfDNA sequencing also revealed antimicrobial resistance gene signatures, enabling early prediction of drug resistance and personalized antibiotic regimens. A prospective multicenter trial involving 120 peritoneal dialysis-associated peritonitis episodes, directly comparing mNGS with conventional culture, reported that mNGS can achieve a sensitivity of 92% versus 68% for culture, reduced the mean time to etiologic diagnosis from 72 to 36 h, and detected atypical pathogens such as Mycobacterium and rare fungi, particularly in antibiotic-pretreated patients [127]. NGS performance was evaluated in 38 peritoneal dialysis-related peritonitis cases, reporting that mNGS nearly doubled pathogen detection rates (84% vs. 42%), demonstrated 90% concordance with antimicrobial susceptibility testing, and prompted treatment modifications in 42% of episodes, underscoring its role in precision antimicrobial stewardship [128].

Moreover, the perception of pathobionts, in addition to mNGS of pathogens, can play a strategic role in the assessment of new, unknown, or poorly characterized microbes. In this regard, the characterization of specific microbiota patterns related to different gut topographies and microbiota diversity through an agnostic approach [129] is revealing the actual microbiota profiles and the deep interplay between the microbiome [130], the host, and sepsis severity outcomes, within a holistic pathophysiological vision of the holobiont. Indeed, trans-kingdom microbial interactions influence the composition of the intestinal microbiota. Microbes and microbial products interact with the host, gaining access to tissues and organs when the integrity of the epithelial barrier is compromised, as occurs with contamination of mesenteric lymph nodes and peritoneal areas [131]. Shifts in gut flora may lead to changes in host–microbe interactions. However, beyond microbial ecology and metataxonomy, microbiota-derived metaproteins and metabolites play a central functional role in modulating microbial metabolism, virulence, and resistance processes. Thus, metaproteomics and metabolomics approaches should be exploited for the full ecological and functional characterization of microbiota communities, providing comprehensive data for the description of deep disease phenotypes, ultimately reinforcing the efficacy of systems medicine in better stratifying patients and optimizing diagnostics, clinical management, and treatment responses [132] without neglecting the exposome as a multifaceted determinant of microbiome variability [133].

Regardless of advanced systems medicine approaches, murine models may still play a central role in assessing the microbe-host interplay. A lipopolysaccharide-induced peritonitis model was performed to characterize host inflammatory cascades, identifying over 4000 differentially expressed genes enriched in NF-κB, MAPK, and cytokine signaling pathways. Subsequent network analysis highlighted hub genes such as Tnf and Il1b [134]. These complementary human and murine omics approaches demonstrate the feasibility of a multi-omic pipeline capable of simultaneously characterizing the peritoneal and peritoneal-associated microbiomes, antibiotic resistance factors, and responses to nutritional and infectious sources of variability, with the ultimate aim of advancing individualized diagnostics, prognostication, and targeted therapeutics in severe secondary peritonitis.

### 8.3. Omics Identification as a Prompt Diagnostic Strategy Tool

Early etiologic classification is pivotal in secondary peritonitis. A pragmatic omics workflow may optimize time-to-result, sharpen source-control decisions, allowing earlier, targeted antimicrobial therapy, especially in culture-negative or antibiotic-pretreated presentations and in PDAP, where timing in catheter salvage versus removal is critical. In prospective PD cohorts, metagenomic NGS and cell-free (cf)DNA sequencing have demonstrated higher sensitivity than culture with earlier pathogen calls, including atypical organisms and resistance determinants, supporting their use for early diagnosis [126,127,128].

Reasonable indications for rapid omics-based diagnosis may include severe sepsis with equivocal source or polymicrobial strains, as well as prior antibiotic exposure with anticipated culture suppression, or again PDAP with high risk of technique failure to inform early catheter removal rather than catheter salvage. Peritoneal fluid and paired plasma might represent eligible biological matrices for cfDNA. Nevertheless, parallel aliquots must be reserved for standard culture according to current guidelines [113]. In addition, sequencing of cfDNA may increase sensitivity in low-volume or partially treated infections, thus revealing possible extra-peritoneal foci seeding the cavity [126].

Metatranscriptomics might enrich for viable organisms and active pathways (i.e., toxins and biofilm programs), aiding distinction between stable colonization and invasive infection. Short-chain fatty acids and β-lactamase activity may be assessed by metabolomics tools adding functional readouts that can support empiric de-escalation or early antifungal treatments in HC-IAI [28,29,30,132].

In stercoraceous peritonitis, omics may confirm early anaerobic bacterial contamination and ESBL risk, driving decisions on carbapenem-sparing or anaerobe-focused regimens after source control. Early detection of *Pseudomonas* spp. or refractory coagulase-negative staphylococci and resistome signals might support catheter removal over salvage in PDAP, aligning with biomarker-guided strategies (NGAL, NLR/PLR/SIRI) [19,20,92,94]. In gastrointestinal perforations with initially low bacterial burden, a negative omics profile coupled with down-trending IL-6/PCT might support short antibiotic courses and relaparotomy [19,97,99].

## 9. Future Directions

Single-cell RNA sequencing (scRNA-seq) has transformed our understanding of peritoneal fibrosis, revealing cellular and molecular signatures predictive of surgical complications. Long-term peritoneal dialysis patients show expansion of cytotoxic CD8^+^ T cells and upregulation of fibrosis-related genes, suggesting these changes could serve as early biomarkers of fibrotic remodeling and adverse outcomes. Although spatial proteomic profiling remains underexplored in abdominal compartment syndrome (ACS), scRNA-seq has identified macrophage subsets (Macro-c2-SSP1, Macro-c5-FCN1&SSP1) and mesothelial cell populations that actively drive fibrosis within the peritoneal cavity [135]. These findings highlight the potential of targeting these specific cell populations through proteomic signatures to enable preemptive clinical interventions, including decompression strategies, before intra-abdominal pressure escalates to critical levels. Organoid technology complements these insights by providing personalized platforms for therapy testing. Organoids retain patient-specific genetic profiles and reproduce drug-response patterns with higher fidelity than conventional two-dimensional cultures. While most applications have focused on epithelial cancers, organoid systems hold promise for evaluating antifibrotic and antifibrinolytic agents in peritoneal fibrosis, enabling accelerated development of patient-tailored therapies [136].

Advances in biofabrication further expand therapeutic possibilities. Melt electro-writing has generated polycaprolactone scaffolds with 30°-angled microfibers coated with primary mesothelial cells. In a mouse model of chemical peritonitis, these scaffolds acted as physical barriers to macrophage infiltration, promoted mesothelial regeneration, and prevented fibrotic adhesion formation, demonstrating their potential as regenerative platforms for integration with future peritoneum-on-chip devices [137].

Integrating scRNA-seq, spatial proteomics, organoids, and biofabrication technologies could transform early detection, mechanistic understanding, and personalized management of peritoneal fibrosis and secondary peritonitis. Future studies should prioritize systematic, variant-specific biomarker discovery, combinatorial therapeutic testing, and translation into clinically actionable strategies. Such multidisciplinary approaches have the potential to deliver precision-guided interventions, reduce morbidity, and improve surgical outcomes in complex abdominal pathologies.

## 10. Conclusions

**Figure 2:**
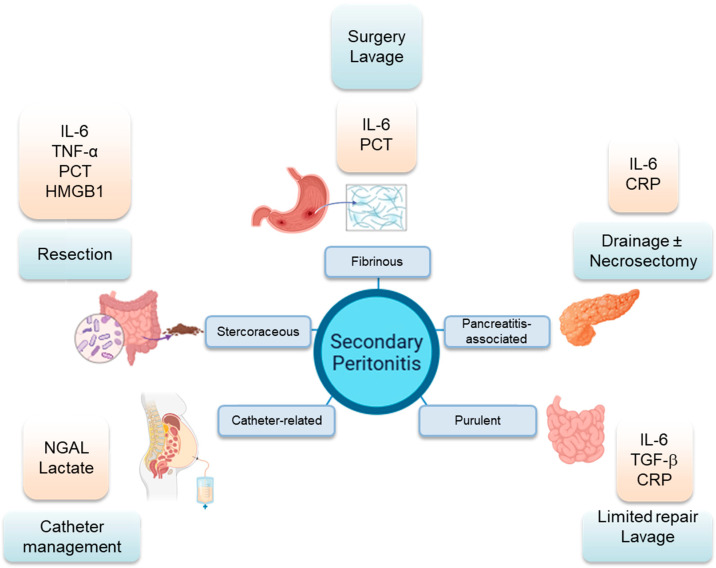
Types of secondary peritonitis, validated biomarkers, and clinical interventions. Orange boxes, known biomarkers; light blue boxes, clinical interventions. CRP, C-reactive protein; HMGB1, high mobility group box 1; IL-6, interleukin-6; NGAL, neutrophil gelatinase-associated lipocalin; PCT, procalcitonin; TGF-β, transforming growth factor β.

In summary, secondary peritonitis encompasses a heterogeneous spectrum of clinical entities: fibrinous, purulent, stercoraceous, pancreatitis-associated, and catheter-related in peritoneal dialysis variants. Despite standardized management protocols, secondary peritonitis continues to carry significant morbidity and mortality. Circulating and compartmentalized biomarkers, including procalcitonin (PCT), interleukin-6 (IL-6), neutrophil gelatinase-associated lipocalin (NGAL), and high-mobility group box 1 (HMGB-1), have provided powerful tools for early diagnosis, prognostication, and antimicrobial stewardship in intra-abdominal infection (Figure 2). Critical knowledge gaps remain, however. The kinetics, threshold values, and cellular origins of these biomarkers across peritonitis subtypes are poorly defined. PCT-guided antibiotic discontinuation can safely shorten treatment in abdominal sepsis, and peritoneal NGAL detects dialysis-associated peritonitis before cultures become positive, yet no systematic comparisons exist across stercoraceous, fibrinous, or chemical variants. Conventional soluble markers also lack spatial resolution, obscuring localized immune microenvironments and tissue-specific injury.

Altogether, these findings highlight the potential for translating systems biology and multi-omics technologies into advanced decision support systems (DSSs) for the personalized management of secondary peritonitis. To this end, systematic biomarker profiling integrating transcriptomics, metagenomics, proteomics and metaproteomics, metabolomics, spatial imaging, and machine-learning approaches remains an essential goal. Validation through standardized protocols, interlaboratory harmonization, and large-scale trials will be required, but the prospect of tailoring interventions to the molecular and cellular signatures of distinct peritonitis subtypes represents a concrete breakthrough toward precision medicine in intra-abdominal infections and inflammation.

## 11. Limitations

Despite the growing body of research, several limitations must be acknowledged. Many of the studies available are preclinical, relying on animal models that may not fully replicate human peritoneal physiology. Current intestinal and peritoneal organoids, as well as peritoneum-on-chip models, yield mechanistic insights but face translational constraints, including incomplete incorporation of immune, neural, and lymphatic elements. Partial mesothelial dedifferentiation and donor drift may undermine chronic remodeling readouts, which in turn may imperfectly correlate with clinical endpoints. Thus, these predictive models risk overfitting and lack external validation. Again, clinical data are often limited to small, single-center observational cohorts, with few randomized controlled trials addressing biomarker-guided strategies in secondary peritonitis. Significant heterogeneity exists in sampling protocols, timing of biomarker measurement, and reporting standards, which hampers cross-study comparison and meta-analysis. Finally, the lack of multicenter validation and standardized thresholds limits the immediate translation of promising biomarkers into clinical practice. Future studies should prioritize large-scale, prospective, and multicenter investigations to establish robust, clinically actionable evidence.

## Figures and Tables

**Table 1:** Major features of clinical variants of secondary peritonitis.

Feature	Fibrinous	Purulent	Stercoraceous	Pancreatitis- Associated	Catheter-Related in Peritoneal Dialysis
**Primary** **contaminant**	Gastric acid, acidic chyme, digestive enzymes	Bile, small bowel content, limited fecal, urine	Gross fecal content	Pancreatic enzymes	Biofilm bacteria
**Microbe type**	Enteric polymicrobial communities	Anaerobes: *Bacteroides fragilis*, *Clostridium perfringens*, *Fusobacterium* spp.; aerobes: *Escherichia coli*, *Klebsiella* spp., *Enterobacter* spp., *Pseudomonas aeruginosa*	Facultative anaerobes: Enterobacteriaceae; anaerobes: *Bacteroides* *fragilis*, *Clostridium* spp., *Fusobacterium* spp.; *Prevotella* spp.	Initially absent followed by presence of opportunistic bacteria: *Acinetobacter* *baumannii*, *Pseudomonas aeruginosa*, *Klebsiella pneumoniae*, *Escherichia coli*	*Staphylococcus* *epidermidis*, *Pseudomonas* *aeruginosa*, *Streptococcus* spp.
**Inflammatory condition**	Variable	Localized, Mild Systemic	Diffuse, Mild-to-Severe Systemic	Protease-driven, Localized	Chronic, low-grade
**Key** **biomarkers**	PCT, IL-6	IL-6, TGF-β, CRP	IL-6, TNF-α, PCT, HMGB1	IL-6, CRP, serum amylase	NGAL, effluent lactate
**Surgical** **approach**	Laparoscopic repair, lavage	Limited repair/lavage	Resection, possible open abdomen	Drainage ± necrosectomy	Catheter salvage vs. removal
**Prognosis**	Variable, timing critical	Variable, generally favorable	High mortality	Moderate, worsens if infected	Technique failure common

**Table 2:** Up-to-date inflammatory Biomarkers in Secondary Peritonitis.

Biomarker	Source	Diagnostic Utility	Prognostic Utility	2025 Update
**PCT**	Serum	PCT for SBP diagnosis in cirrhosis shows 76% sensitivity and 87% specificity [83]	PCT-guided antibiotic discontinuation reduces antibiotic duration by 1.9 days without increasing mortality or relapse risk [84]	Meta-analysis of 5 RCTs shows 15% reduction in antibiotic days without mortality increase [85].
**IL-6**	Serum, peritoneal fluid	Early rise (<6 h) correlates with contamination load [86]	Dialysate IL-6 as a predictor of peritonitis in patients on peritoneal dialysis	IL-6 as biomarker of PDAP severity [87,88]
**HMGB1**	Serum	Reflects cellular necrosis; higher in stercoraceous cases [89,90]	Associated with ICU length of stay [40]	scRNA-seq reveals HMGB1+ macrophage subset [91]
**NGAL**	Peritoneal fluid	Detects PD-associated infection earlier than culture [92]	In PD distinguishes cases of peritonitis from non-infected cases with high sensitivity and specificity, preceding microbiological confirmation [93]	Concentrations >250 ng/mL double the risk of treatment failure in peritonitis-associated catheter removal [93]
**NLR**	Peripheral Blood	Simple, low-cost severity marker [94]	NLR shows an incremental relationship with the risk of treatment failure (OR 1.82) [19]	Use of NLR, PLR, and SIRI levels for enhancing PDAP diagnostic accuracy [19,20,94]
**miR-233**	Peritoneal fluid	Severity marker in PD	Predicts catheter loss	Correlates with NGAL levels [95]
**miR-432-5p**	Peritoneal fluid exosomes	biomarker of impaired fluid and sodium removal in PD	Predictive of disease severity	miR-432-5p inhibits α-ENaC expression in mesothelial cells [96].

RCTs, randomized controlled trials; ICU, intensive care unit; neutrophil-to-lymphocyte ratio, NLR; PLR, platelet-to-lymphocyte ratio; systemic immune-inflammation index, SIRI; PD, peritoneal dialysis; PDAP, peritoneal dialysis-associated peritonitis.

## Data Availability

No new data were created for this Review article.

## Abbreviations

The following abbreviations are used in this manuscript:

3′-UTR	three prime untranslated region
5′-UTR	five prime untranslated region
AUC	area under the curve
CCL2	chemokine (C-C motif) ligand 2
CLP	cecal ligation and puncture
CXCL1	chemokine (C-X-C motif) ligand 1
ECM	extracellular matrix
ESBL	extended-spectrum beta-lactamase
EPS	encapsulating peritoneal sclerosis
G-CSF	granulocyte colony-stimulating factor
HC-IAI	health care-associated intra-abdominal infections
HMGB1	high mobility group box 1
ICU	intensive care unit
IKK	IκB kinase
IL-6	Interleukin-6
IL-8	Interleukin-8
LPS	lipopolysaccharide
MCP-1	monocyte chemoattractant protein-1
MMP-9	matrix metalloproteinase-9
MMT	mesothelial-to-mesenchymal transition
NF-kB	nuclear factor kappa-light-chain-enhancer of activated B cells
NGAL	neutrophil gelatinase-associated lipocalin
NLR	neutrophil-to-lymphocyte ratio NGAL
NLRP3	NLR family pyrin domain containing 3
NOD1/2	nucleotide-binding oligomerization domain-containing protein 1/2
PCI	peritoneal contamination and infection
PCT	procalcitonin
PD	peritoneal dialysis
PDAP	peritoneal dialysis-associated peritonitis
PICU	pediatric intensive care unit
PLR	platelet-to-lymphocyte ratio
PUP	peptic ulcer perforation
RCT	randomized controlled trial
SAP	severe acute pancreatitis
SIRI	systemic immune-inflammation index
TGF-β	transforming growth factor β
TLR-4	toll-like receptor 4
TNF-α	tumor necrosis factor α
